# Safety and Effectiveness of* Mist Antiaris*, a Herbal Preparation for Treatment of Peripheral Neuropathy

**DOI:** 10.1155/2019/2607872

**Published:** 2019-07-24

**Authors:** S. Antwi, J. Asiedu-Larbi, O. N. K. Martey, O. Quasie, M. Boakye-Yiadom, F. Ayertey, R. Yeboah, C. A. Sapaty, D. Offei-Abrokwa, D. Oduro-Mensah, E. K. Kumatia, A. Ocloo

**Affiliations:** ^1^Pharmacology and Toxicology Department, Centre for Plant Medicine Research, P. O. Box 73, Mampong, Akuapem, Ghana; ^2^Clinical Research Department, Centre for Plant Medicine Research, P. O. Box 73, Mampong, Akuapem, Ghana; ^3^Department of Applied Chemistry and Biochemistry, Faculty of Applied Sciences, University for Development Studies, Navrongo Campus, P. O. Box 24, Navrongo, Ghana; ^4^Phytochemistry Department, Centre for Plant Medicine Research, P. O. Box 73, Mampong, Akuapem, Ghana; ^5^Department of Biochemistry, Cell and Molecular Biology, University of Ghana, P. O. Box LG 54, Legon, Ghana

## Abstract

Mist Antiaris is a herbal decoction for treatment of nervous disorders. Safety and efficacy were evaluated in Sprague-Dawley rats and human patients, respectively. Acute toxicity was assessed by administration of a single 5000 mg/kg oral dose of decoction to a group of six rats. For subchronic toxicity, four groups of six rats each received water (control) or 10, 100, or 200 mg/kg oral doses of decoction daily for eight weeks. Body weight, serum, urine, and hematological profile of the animals in each group were monitored over the period. Effects of treatment on pentobarbital-induced sleeping time and histology of liver, lung, heart, and kidney tissue were assessed at the end of the study. There was no evidence of acute toxicity within 48 hours of the oral dose. Over the 8-week period, body weight increases in Mist Antiaris treatment groups were reduced relative to the control group. There were no significant differences in urine profile, serum biochemistry, hematological parameters, and pentobarbital-induced sleeping time. Tissue histology revealed no differences relative to controls. Assessment of efficacy was by retrospective review of data on patients who presented with peripheral neuropathy. Treatment resulted in 53.7 % of patients reporting complete resolution and 15.7 % showing reduction in neuropathic symptoms. The data demonstrate that there is no toxicity due to subchronic administration of Mist Antiaris in Sprague-Dawley rats. The reduction or resolution of neuropathic symptoms indicated by patents' file data provides evidence to suggest that* Mist Antiaris* has antineuropathic effects.

## 1. Introduction

Peripheral neuropathy is a long-term complication that involves one or several nerve trunks causing pain, numbness, tingling and burning sensation, muscle weakness, and/or atrophy. It is a common neurological presentation at many Outpatient Clinics in Ghana [1]. Globally, peripheral neuropathy is reported to affect 50% of diabetes patients, with type II diabetics accounting for approximately 45% and type I accounting for 54% of recorded cases [2]. Incidence of peripheral neuropathy among hypertensive individuals is approximately 97.2% [3] and is likely to double in some developing countries by 2030 [2]. Other conditions such as leprosy, HIV infection, alcohol abuse, and aging are associated with clinical cases of peripheral neuropathy, most occurring within the age range of 50 - 69 years, especially in females [4, 5]. Commonly affected parts of the body are the upper and lower extremities [2, 6].

Mist Antiaris is a monoherbal decoction from the Center for Plant Medicine Research (CPMR), Mampong-Akuapem, Ghana, prepared from the stem bark of* Antiaris africana*.* A. africana *is a large, deciduous tree from the family Moraceae. Extracts from the stem bark and other parts of* A. africana* have been used for treatment of sore throat, leprosy, rheumatic pain, and neurological disorders such as seizures and chronic tremor of the limbs [7]. Compounds that have been isolated from the bark include flavonoids, betulinic acid, ursolic acid, oleanolic acid, strophanthidol, periplogenin, methyl strophanthinate, and a *γ*-lactone named antialactone, some of which have been shown to possess antibacterial, antioxidant, and/or antiproliferative activities [8–11].

Although Mist Antiaris has been administered by the Clinic Department of CPMR for over two decades as treatment for nervous disorders, not only has its safety not been fully authenticated in preclinical studies but also the clinical outcome of its use has not been validated. This work was aimed at evaluating the safety of Mist Antiaris in Sprague-Dawley rats, and to validate its clinical use in the management of peripheral neuropathy.

## 2. Materials and Methods

### 2.1. Materials

#### 2.1.1. Chemicals, Reagents, and Test Kits

Aspartate aminotransferase (AST), alkaline phosphatase (ALP), alanine aminotransferase (ALT), urea, creatinine, and Creatinine Kinase MB (CK-MB) were purchased from ELITech Clinical System (Sees, France). Urine test strips (UroColor™ 10) were supplied by Standard Diagnostics Inc. (Kyonggi-do, Korea). Xylene and pentobarbital were obtained from Sigma-Aldrich (St. Louis, MO). All other chemicals were purchased from British Drug Houses (BDH) Ltd. (Poole, UK).

#### 2.1.2. Animals

Male Sprague-Dawley rats (SDRs), weighing between 200 and 230 g, were obtained from the Animal Experimentation Unit of CPMR. The rats had free access to pelleted feed obtained from AGRICARE Ltd., Kumasi, with free access to sterilized distilled water and kept under a 12-hour light-dark cycle. The animals were handled in accordance with National Institute of Health Guidelines for the Care and Use of Laboratory Animals (NIH Publication no. 85-23, 1985). All animal experiments and review of clinical data were approved by the Ethics Committee of Centre for Plant Medicine Research (CPMR/PT/CL /2016-06/004).

#### 2.1.3. Mist Antiaris

Mist Antiaris was obtained from the Production Department of CPMR. The decoction was lyophilized to obtain a powder which was stored at 4°C until use.

### 2.2. Methods

This study consisted of a preclinical study using Sprague-Dawley rats and analysis of a retrospective clinical data using folders of patients attending a neuropathy clinic at the CPMR.

#### 2.2.1. Preclinical Study

(*1) Acute Toxicity*. A single oral dose (up to 2 ml/rat) of the lyophilized product, reconstituted in distilled water, was administered by oral gavage at 5000 mg per kg of body weight to a group of six rats after an overnight fast. Animals in the control group received sterile distilled water. Mortality and general behavior of the animals were observed over a 48-hour period. Surviving animals were observed for a further period of 12 days for symptoms of toxicity, such as piloerection and defects in lachrymatory, locomotory, and respiratory activities.

(*2) Subchronic Toxicity*. Twenty-four male SDRs were put in four groups (six rats per group) and kept in separate metal cages. Group 1 was kept as control and received sterile distilled water by oral gavage for eight weeks. Groups 2, 3, and 4 received daily oral doses of 10, 100, or 200 mg/kg body weight of Mist Antiaris, respectively, each representing the therapeutic dose, 10 times, or 20 times the therapeutic dose. Body weight of each animal was recorded on day zero (baseline) and weekly thereafter.

(*a) Urinalysis*. Urine samples from rats in each treatment group were collected by involuntary discharge on day zero (baseline), and after four and eight weeks. Samples were analyzed using urine reagent strips UroColor™ 10 (Standard Diagnostic Inc., Korea). 

(*b) Blood Sampling*. Blood samples of the rats in each treatment group were obtained by tail bleeding on day zero (baseline), and after four and eight weeks into Eppendorf tubes without anticoagulant, centrifuged at 3000 rpm for 5 min (Denley BS 400, England), and the serum obtained was stored at -40°C pending use for biochemical analyses. Other blood samples were collected into EDTA-coated tubes (Zhejiang Guangdong Medical Technology Co. Ltd) and used for hematological analysis within 24 hours. 

(*c) Serum Biochemical Analysis*. Levels of AST, ALP, ALT, CK-MB, creatinine, and urea in serum samples prepared from rats' blood samples were determined using protocols from ELITech Clinical System (Sees, France) with a semiautomated clinical chemistry analyzer, Microtech–3000 (Vital Scientific N.V, The Netherlands). 

(*d) Hematological Analysis*. Red blood cells (RBC), white blood cells (WBC), mean corpuscular volume (MCV), hematocrit (HCT), hemoglobin (HGB), platelet (PLT), mean corpuscular hemoglobin (MCH), mean platelet volume (MPV), thrombocytes (PCT), red cell distribution width (RDW), lymphocytes(LYMPH), and mean corpuscular hemoglobin concentration (MCHC) were determined with a Sysmex Haemanalyser (Japan). 

(*e) Pentobarbital-Induced Sleeping Time*. Pentobarbital-induced sleeping time was determined at termination of administration of the decoction. An intraperitoneal injection of pentobarbital (40 mg/kg) in normal saline was administered to all experimental rats. The time taken for animals to completely lose their reflexes was noted as sleep time and the time they completely regained their righting reflexes, as wake time. The difference between the two was noted as the sleeping time. 

(*f) Organ Wet Weight and Histology*. At the end of the eight weeks (termination), rats in each group were euthanized by cervical dislocation, and the spleen, heart, kidney, liver, and lungs were excised and weighed. For each animal, mean organ wet weight to body weight ratio of selected organs was determined. Storage of excised organs was in 10% neutral buffered formalin. The organs were dehydrated with ethanol, cleared with chloroform, impregnated with paraffin wax, and embedded in paraffin wax. Tissue sections (5 *μ*m) of the embedded organs were mounted on glass slides, stained with hematoxylin and eosin dyes, and examined by light microscope (Dialux 22; Leitz, Wetzlar, Germany).

#### 2.2.2. Retrospective Case Study

(*1) Sample Size Justification*. This study involved secondary data of all patients who presented at the Clinic of CPMR with peripheral neuropathy, between December 2015 and December 2016. An overall of 152 folders were retrieved and a selection was made based on the following inclusion criteria: a patient (1) must have presented at least three of the symptoms of peripheral neuropathy, such as numbness, burning sensation, pain, and cramps in the periphery, (2) must not have been on any allopathic treatments such as antidepressants, antiseizure medication, antipsychotic agents, analgesics, or any herbal supplement(s) for a month prior to first visit, and (3) must have reported for review at least thrice over the treatment period. The presence of three symptoms of peripheral neuropathy was scored plus (+) as present and nil (0) as absent. Persistence or resolution of symptoms was tracked. Patients were scored under three categories, namely, absence of neuropathy symptoms, reduction in neuropathy symptoms, and no improvement in neuropathy symptoms.

(*2) Intervention*. Data retrieved indicated that patients received 60 ml Mist Antiaris thrice daily. The product was prepackaged and dispensed in a 330 ml amber plastic bottle. Typically, treatment was given to last for at least three months. 

(*3) Study Limitations*. The number of clients used in the study was limited due to dropouts as a result of failure of patients to return for review, default in medication usage, and failure to visit on due review dates

### 2.3. Statistical Analysis

For the preclinical studies, one-way analysis of variance (ANOVA) followed by the Newman-Keuls post hoc test was conducted to determine statistical significance. All significance analyses were conducted at 95 % significance level. All statistical tests and graphs were obtained with GraphPad prism version 5. Clinical data were analyzed using the IBM Statistical Package for Social Sciences (SPSS) 17. Age was summarized as group data with an interval of 6 years.

## 3. Results

### 3.1. Preclinical Study

#### 3.1.1. Acute Toxicity Studies

Administration of a single oral dose of Mist Antiaris (5000 mg/kg) did not result in any mortality within 48 hours. During observation period of additional 12 days, no differences with respect to respiration, locomotion, lachrymation, or piloerection were observed between the treated animals and the control group.

#### 3.1.2. Subchronic Toxicity Studies

**Figure 1:**
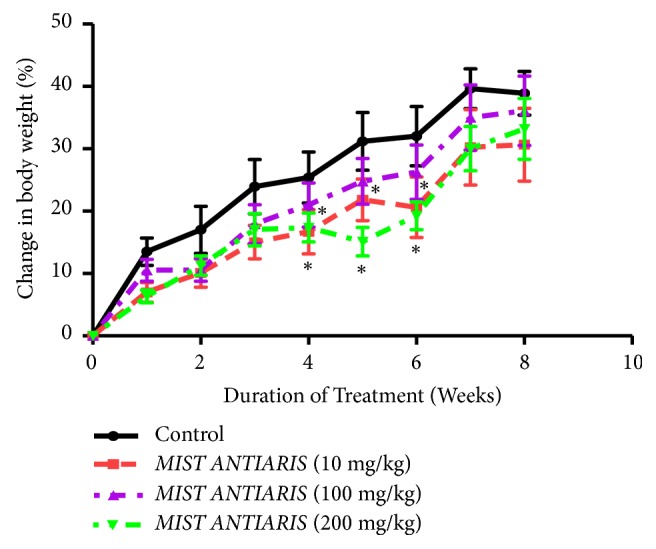
Percentage change in mean body weight of SDRs treated with Mist Antiaris over a period of eight weeks. Each point represents mean ± S.E.M. of n = 6. ^*∗*^Values significantly different from the control.

(*1) Mean Body Weight*. Over the 8-week treatment period, percent mean body weight of rats in the control and treatment groups increased significantly (p < 0.05), albeit a lesser extent in the treatment groups (Figure 1).

**Figure 2:**
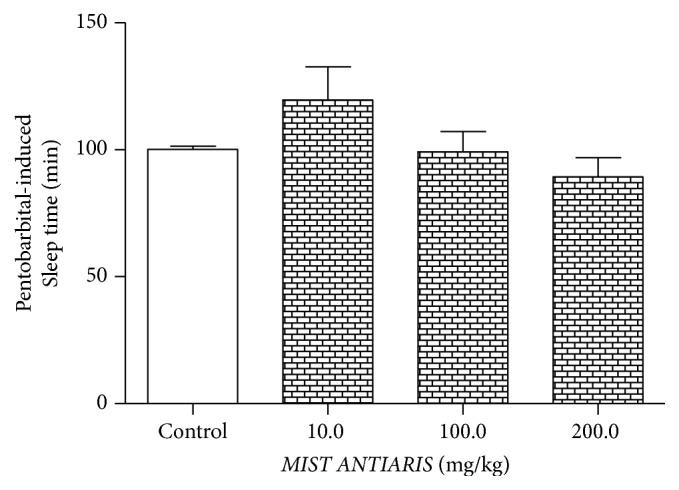
Effect of the treatment of Mist Antiaris on the pentobarbital-induced sleeping time of SDRs.

**Figure 3:**
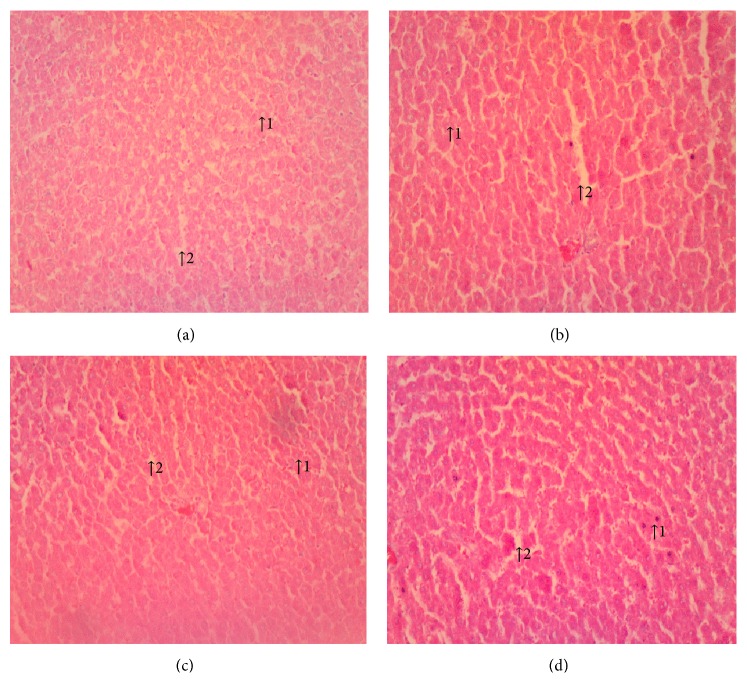
Micrographs of the liver at termination of treatment for control (a), therapeutic dose (b), 10X the therapeutic dose (c), and 20X the therapeutic dose (d) of Mist Antiaris showing normal hepatocytes (1) and interstitial spaces (2). Magnification: x 100.

**Figure 4:**
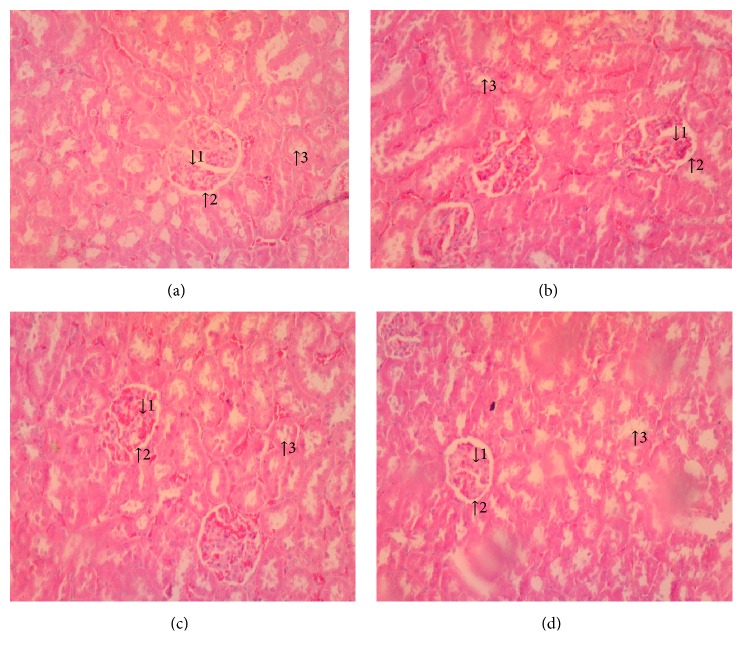
Micrographs of the kidney at termination of treatment for control (a), therapeutic dose (b), 10X therapeutic dose (c), and 20X therapeutic dose (d) of Mist Antiaris, showing no difference in the appearance of glomerulus (1), Bowman's capsule (2), and renal tubules (3). Magnification: x 100.

**Figure 5:**
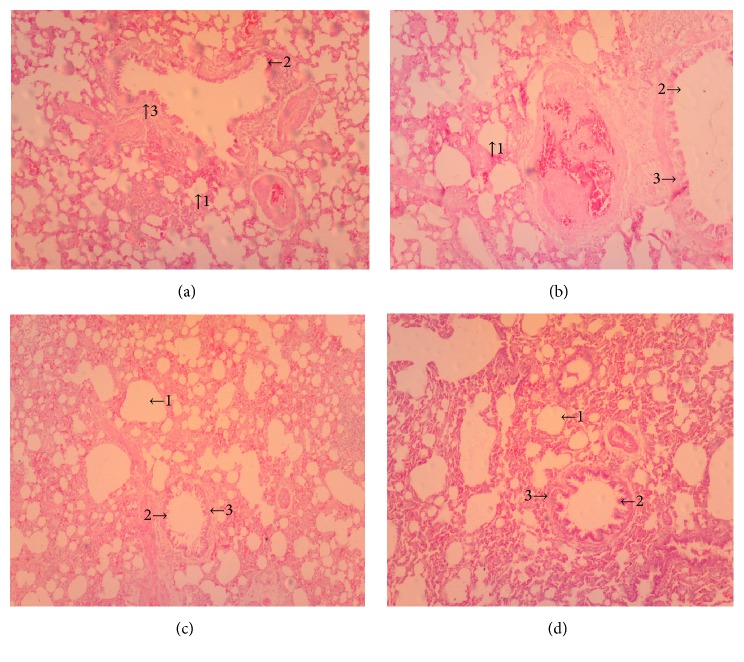
Micrographs of the lung at termination of treatment for control (a), the therapeutic dose (b), 10X the therapeutic dose (c), and 20X the therapeutic dose (d) of Mist Antiaris, showing normal alveolar areas (1), Clara cells (2), lining the normal bronchiolar epithelial wall (3). Magnification: x 100.

**Figure 6:**
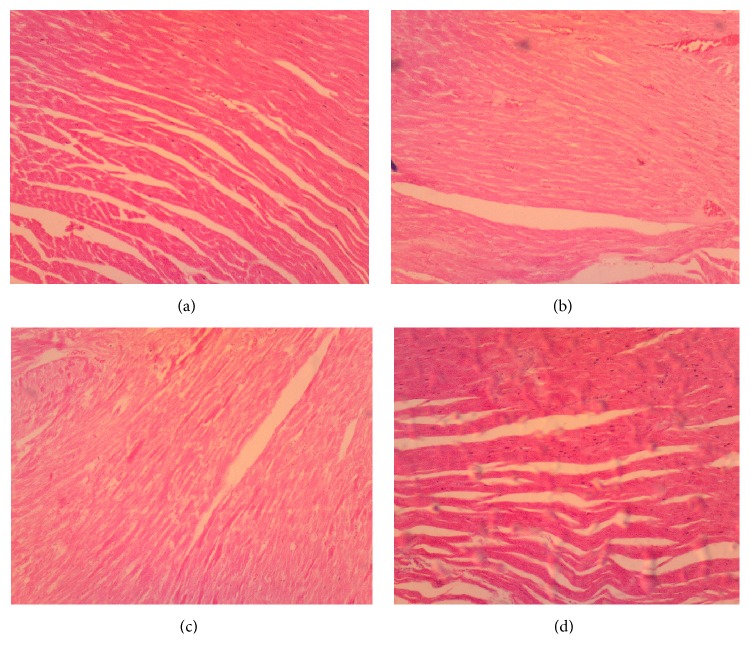
Micrographs of the heart muscle of animals at termination of treatment for control (a), therapeutic dose (b), 10X the therapeutic dose (c), and 20X the therapeutic dose (d) of Mist Antiaris, showing no differences in morphology of cardiac muscles. Magnification: x 40.

There were significant reductions in body weight in the 10 mg/kg and 200 mg/kg treatment groups compared to the control group between the 4^th^ and 6^th^ weeks; however these reductions were leveled off after the 6^th^ week.

(*2) Mean Organ Wet Weight and Urinalysis*. The data did not show any significant differences in mean organ wet weight values (Table 1) or parameters for urinalysis (Tables 2 and 3) between the control group and any of the Mist Antiaris treatment groups. 

(*3) Serum Biochemistry and Hematology*. Relative to the control group, significant differences (p < 0.05) in ALP activity were observed for the therapeutic and 10X therapeutic dose groups (Tables 4 and 5). Differences in AST, ALT, CK-MB, creatinine, and urea levels were not significant. Data from hematological analyses did not indicate any significant differences between the control group and Mist Antiaris treatment groups (Tables 6 and 7). 

(*4) Pentobarbital-Induced Sleeping Time*. Mist Antiaris-treated rats recorded dose-dependent, although insignificant, decreases in sleeping time compared to the control group (Figure 2). 

(*5) Histopathology*. Histopathological examination of liver, kidney, lung, and heart tissue did not reveal any differences between control and Mist Antiaris-treated rats (Figures 3–6).

### 3.2. Retrospective Clinical Study

#### 3.2.1. Demographics

Of 120 patients whose data were reviewed in the study, 82 (68.3%) were female while 38 (31.7%) were male (Table 8). Approximately 52 % of participants were in the age range of 50-65 years (Table 9). The most significant comorbidities associated with patients sampled were hypertension and Type 2 diabetes mellitus which together were recorded in 80% of participants.

#### 3.2.2. Peripheral Neuropathy Status

**Figure 7:**
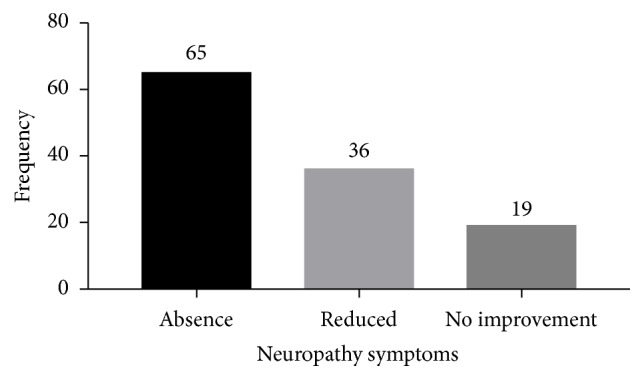
Peripheral neuropathy status of participants.

After the 6-week course of treatment with Mist Antiaris, complete resolution of symptoms of peripheral neuropathy was reported by 65 (53.7%) subjects, 36 (29.8%) subjects reported reduction in symptoms, and 19 (15.7%) had no improvement (Figure 7).

## 4. Discussion

Mist Antiaris is a monoherbal herbal preparation produced in Ghana for the management of nervous disorders, including epilepsy. The single oral dose of Mist Antiaris administered did not cause any mortality within 48 hours, suggesting that the oral median lethal dose (LD_50_) is greater than 5000 mg/kg. The absence of physical signs of toxicity as evidenced by normal respiratory, locomotory, and lachrymatory activities, as well as the absence of piloerection, further indicated that Mist Antiaris at 500X the therapeutic dose (5000 mg/kg) may not affect physiological, behavioral, and autonomic profiles [12].

At the end of the eight-week treatment period, body weights of animals did not differ significantly between the control and treatment groups (Figure 1). Wet weights of the heart, kidneys, liver, lungs, and spleen of animals in the treatment groups were not different from the control group (Table 1), giving a strong indication of the absence of organ-specific toxicity. This observation was supported by the absence of differences between micrographs of selected tissue sections of representative animals from each group (Figures 3–6).

Serum concentrations of creatinine and urea were not affected by the herbal decoction at the end of the experimental period (Table 5), suggesting the absence of renal impairment in the rats. Kidney micrographs (Figure 4) and the results of urinalysis (Table 3) further give credence to this finding.

Damaged liver cells release intracellular enzymes such as, ALT, AST, and ALP into the blood resulting in an elevation in their activities. These elevations can, thus, be used as nonspecific indices of hepatotoxicity [13]. The fact that the herbal extract Mist Antiaris did not alter the serum activities of the aforementioned parameters (Tables 4 and 5) indicates that liver function was not compromised in the experimental animals during the treatment period. This assertion is further supported by liver micrographs of the histopathological examination which showed normal hepatocytes (Figure 4).

The serum activity of CK-MB, normally used as an index of cardiotoxicity, was not affected in the treated rats (Tables 4 and 5), ruling out the development of cardiotoxicity. Micrographs showing normal heart muscle cells (Figure 6) further corroborate this claim. Similar observations were made for the lungs (Figure 5) and spleen (result not shown).

The above observations are corroborated in a similar study by Ilesanmi* et al*., [14] which showed the methanolic extract of* A. africana* to have nontoxic effects on liver, kidney, and heart markers of toxicity at doses below 200 mg/kg, in Wistar rats.

Subchronic treatment of the experimental animals with Mist Antiaris did not affect any of the haematological indices measured, suggesting the absence of types of haematological toxicity.

The hypnotic drug, Pentobarbital, is metabolized by the cytochrome P450 isozyme system [15], so its metabolism by the liver is assessed in drug interaction studies, by determining the effect on pentobarbital-induced sleeping time [16]. The fact that pentobarbital-induced sleeping time for all the treatment groups was similar to the values obtained for the controls is an indication that the plant preparation neither induced nor inhibited isozymes of the CYP450 system.

From the clinical retrospective study, treatment of patients with Mist Antiaris for a period of six weeks resulted in different degrees of amelioration of neuropathic symptoms. This observation was demonstrated with a reduction in the frequency of reports of neuropathic symptoms by patients, indicating that Mist Antiaris may possess some antineuropathic properties. In the current study, neuropathic symptoms were more prevalent in female participants (68.3 %) and the elderly between ages 60 and 80 (53%). This is in agreement with a cross-sectional study by Asad* et al*. [2] which showed that risk factors contributing to neuropathy included gender and age.

## 5. Conclusion

This study has shown that the oral LD_50_ of ‘Mist Antiaris' in male adult Sprague-Dawley rats (SDRs) is greater than 5000 mg/kg. There appears to be no overt specific toxicity following subchronic administration of ‘Mist Antiaris' at the TP dose (10 mg/kg), 10 times the TP dose (100 mg/kg), and 20 times the TP dose (200 mg/kg) as evidenced by the results of biochemical, hematological, and urine analysis in rats. In addition, the herbal extract may not cause any drug-herb or drug-drug interaction. For the retrospective study, treatment of patients with neuropathy resulted in a reduction or absence of neuropathic symptoms after three months of treatment implying that Mist Antiaris may possess some antineuropathic properties. It is recommended that a prospective cohort study and a controlled clinical trial should be undertaken to confirm the safety and effectiveness of the product in humans.

## Figures and Tables

**Table 1:** Mean wet organ/body weight at termination of treatment with Mist Antiaris.

	Organ weights/weight (%)			
	Treatment Groups			
Organ	Control	Mist Antiaris		
		10 mg/kg	100mg/kg	200 mg/kg
Liver	2.88 ± 0.08	3.03 ± 0.03	3.08 ± 0.12	2.91 ± 0.11
Kidneys	0.54 ± 0.02	0.58 ± 0.02	0.58 ± 0.02	0.57 ± 0.02
Heart	0.29 ± 0.01	0.29 ± 0.01	0.32 ± 0.30	0.30 ± 0.01
Lungs	0.60 ± 0.05	0.60 ± 0.06	0.51 ± 0.06	0.62 ± 0.06
Spleen	0.23 ± 0.01	0.22 ± 0.01	0.24 ± 0.01	0.22 ± 0.01

Values are Mean ± SEM, n = 6

**Table 2:** Effect of Mist Antiaris on urine parameters after four weeks of treatment.

Parameters	Treatment Group			
	Control	Mist Antiaris		
		10 mg/kg	100mg/kg	200 mg/kg
Urobilinogen (mg/dl)	N	N	N	N
Glucose (mg/dl)	-	-	-	-
Bilirubin (mg/dl)	-	-	-	-
Ketones (mg/dl)	±	±	±	±
S.G.(g/ml)	1.012	1.018	1.022	1.015
Blood (RBC/*µ*l)	-	-	-	-
pH	6.5	6.7	6.0	6.5
Protein (g/l)	-	-	-	-
Nitrite	-	-	-	-
Leukocytes (WBC/*µ*l)	-	-	-	-

-: absent, N: normal, ±: trace

**Table 3:** Effect of Mist Antiaris on urine parameters after eight weeks of treatment.

Parameters	Treatment Group			
	Control	Mist Antiaris		
		10 mg/kg	100mg/kg	200 mg/kg
Urobilinogen (mg/dl)	N	N	N	N
Glucose (mg/dl)	-	-	-	-
Bilirubin (mg/dl)	±	±	-	-
Ketones (mg/dl)	±	±	±	±
S.G.(g/ml)	1.036	1.022	1.021	1.020
Blood (RBC/*µ*l)	-	-	-	-
pH	6.60	6.10	6.00	5.90
Protein (g/l)	-	-	-	-
Nitrite	-	-	-	-
Leukocytes (WBC) *µ*l	-	-	-	-

-: absent, N: normal, ±: trace

**Table 4:** Serum biochemical analysis after four weeks of treatment with Mist Antiaris.

Parameter	Treatment Groups			
	Control	Mist Antiaris		
		10 mg/kg	100 mg/kg	200 mg/kg
AST (U/L)	141 ± 24.1	168 ± 18.2	155 ± 9.51	144 ± 11.1
ALP (U/L)	366 ± 27.6	427 ± 31.8^*∗*^	469 ± 32.6^*∗*^	405 ± 54.7
ALT (U/L)	37.6 ± 6.09	34.0 ± 3.20	27 ± 4.52	28.3 ± 3.49
CK-MB (x10^1^U/L)	175 ± 19.2	199 ± 22.6	145± 15.1	144 ± 13.2
Creatinine (*µ*mol/l)	36.6 ± 0.89	31.9 ± 3.37	32.4 ± 4.76	28.3 ± 2.01
Urea (mmol/l)	7.47 ± 0.67	6.33 ± 0.36	6.94 ± 0.51	6.71 ± 0.51

Values are Mean ± SEM, n = 6

**Table 5:** Serum biochemical analysis after eight weeks of treatment with Mist Antiaris.

Parameter	Treatment Groups			
	Control	Mist Antiaris		
		10 mg/kg	100mg/kg	200 mg/kg
AST (U/L)	160 ± 7.53	187 ± 15.3	163 ± 7.50	200 ± 12.5
ALP (U/L)	410 ± 48.0	474 ± 40.0^*∗*^	549 ± 32.4^*∗*^	453± 52.0
ALT (U/L)	67.5 ± 9.34	55.7 ± 5.36	38.8 ± 2.28	36.2 ± 2.96
CK-MB (x 10^1^ U/L)	185 ± 34.2	180 ± 40.4	174 ± 17.4	167 ± 32.3
Creatinine (*µ*mol/l)	63.0 ± 2.01	54.9 ± 1.90	58.3 ± 2.54	61.5 ± 0.70
Urea (mmol/l)	6.04 ± 0.50	6.13 ± 0.02	6.45 ± 0.67	6.23 ± 0.51

Values are Mean ± SEM, n = 6

**Table 6:** Effect of Mist Antiaris on hematological parameters after four weeks of treatment.

Parameters	Treatment Group			
	Control	Mist Antiaris		
		10 mg/kg	100 mg/kg	200mg/kg
WBC (x10^3^ /*µ*L)	14.9 ± 1.09	13.1 ± 1.25	14.4 ± 0.77	14.2 ± 1.12
MPV (f L)	7.20 ± 0.09	7.27 ± 0.08	7.08 ± 0.07	7.26 ± 0.07
RBC (x10^6^ /*µ*L)	7.79 ± 0.17	8.15 ± 0.08	8.14 ±0.20	8.20 ± 0.17
Hb (g/ d L)	15.0 ± 0.17	15.0 ± 0.23	15.0 ± 0.32	15.3 ± 0.18
HCT (%)	47.4 ± 0.63	47.8 ± 0.88	48.3 ± 1.23	49.0 ± 0.60
MCV (f L)	60.9 ± 1.04	58.7 ± 0.76	59.4 ± 0.35	59.8 ± 0.84
MCH (pg.)	19.3 ± 0.47	18.4 ± 0.20	18.5 ± 0.13	18.7 ± 0.29
MCHC (g/ d L)	31.7 ± 0.34	31.4 ± 0.15	31.1 ± 0.18	31.3 ± 0.15
PLT (x10^3^/ *µ*L)	875 ± 28.8	848 ± 80.8	912 ± 27.3	899 ± 31.2
LYM %	73.8 ± 1.64	73.0 ± 2.27	73.9 ± 3.26	76.8 ± 1.52
LYM #	10.9 ± 0.63	9.65 ± 0.97	10.2 ± 0.42	10.9 ± 0.79
RDW (f L)	31.4 ± 0.32	30.1 ± 0.58	30.6 ± 0.41	30.8 ± 0.52

Values are Mean ± SEM, n = 6

**Table 7:** Effect of Mist Antiaris on hematological parameters after eight weeks of treatment.

Parameters	Treatment Group			
	Control	Mist Antiaris		
		10 mg/kg	100 mg/kg	200 mg/kg
WBC (x10^3^ /*µ*L)	18.5 ± 1.09	18.4 ± 1.27	18.2 ± 1.09	19.1 ± 1.39
MPV (f L)	7.30 ± 0.12	7.26 ± 0.03	7.03 ± 0.05	6.95 ± 0.04
RBC (x10^6^ /*µ*L)	7.14 ± 0.21	7.41 ± 0.19	7.73 ± 0.12	7.51 ± 0.15
Hb (g/ d L)	13.3 ± 0.26	13.1 ± 0.29	13.8 ± 0.12	13.5 ± 0.22
HCT (%)	42.8 ± 0.66	43.1 ± 0.68	44.4 ± 0.53	43.6 ± 0.58
MCV (f L)	60.0 ± 1.12	58.3 ±0.25	57.5 ± 0.28	58.2 ± 0.63
MCH (pg.)	18.6 ± 0.50	17.8 ± 0.25	17.9 ± 0.17	18.0 ± 0.24
MCHC (g/ d L)	31.1 ± 0.17	30.6 ± 0.23	31.1 ± 0.17	31.0 ± 0.14
PLT (x10^3^/ *µ*L)	864 ± 74.8	911 ± 56.0	993 ± 51.0	939 ± 37.5
LYM %	70.9 ± 1.63	70.3 ± 4.95	75.7 ± 1.25	68.5 ± 4.40
LYM #	13.0 ± 0.75	12.8 ± 1.03	13.7 ± 0.76	12.9 ± 0.83
RDW (f L)	31.1 ± 0.47	30.0 ±0.38	30.7 ± 0.44	31.9 ± 0.97

Values are Mean ± SEM, n = 6

**Table 8:** Sex distribution of participants.

Sex	Frequency	Percent
Male	38	31.7
Female	82	68.3
Total	120	100.0

**Table 9:** Age Groupings for Participants.

Age Groups	Frequency	Percent
39-45	4	3.3
46-52	18	14.9
53-59	36	29.8
60-66	27	22.3
67-73	23	19.8
74-80	12	9.9
Total	120	99.2

## Data Availability

The data used to support the findings of this study are available from the corresponding author upon request.
